# Models of Cochlea Used in Cochlear Implant Research: A Review

**DOI:** 10.1007/s10439-023-03192-3

**Published:** 2023-04-22

**Authors:** Filip Hrncirik, Iwan Roberts, Ilkem Sevgili, Chloe Swords, Manohar Bance

**Affiliations:** 1 Cambridge Hearing Group, Cambridge, UK; 2 Department of Clinical Neurosciences, University of Cambridge, Cambridge, CB2 0QQ UK; 3 Department of Physiology, Development and Neurosciences, University of Cambridge, Cambridge, CB2 3DY UK

**Keywords:** Cochlear implants, Cochlea anatomy, Cochlea models, Additive manufacturing

## Abstract

As the first clinically translated machine-neural interface, cochlear implants (CI) have demonstrated much success in providing hearing to those with severe to profound hearing loss. Despite their clinical effectiveness, key drawbacks such as hearing damage, partly from insertion forces that arise during implantation, and current spread, which limits focussing ability, prevent wider CI eligibility. In this review, we provide an overview of the anatomical and physical properties of the cochlea as a resource to aid the development of accurate models to improve future CI treatments. We highlight the advancements in the development of various physical, animal, tissue engineering, and computational models of the cochlea and the need for such models, challenges in their use, and a perspective on their future directions.

## Introduction

The unique spiral-structure of the cochlea is essential to its function as the hearing sensory organ. It transduces physical fluid pressure waves into neural impulses that can be interpreted by the brain to sense and understand the acoustic environment. Although this shell-like structure is vital to one of our most essential senses, relatively little has been done to manufacture an artificial cochlea. In vivo experiments have provided a wealth of information about the mechanisms of hearing [9, 52, 57, 125]; however, artificial models could provide a platform to further understand and address many of the remaining challenges in repairing hearing impairments.

Hearing impairment is the most prevalent sensory deficit in humans, affecting 466 million people worldwide (World Health Organisation) [153]. Cochlear implants (CIs) have been transformative for those suffering from severe-to-profound hearing loss by bypassing normal acoustic hearing mechanisms and directly stimulating the cochlear nerve electrically. However, key limitations still constrain the clinical effectiveness and wider eligibility of these implants. Mechanical trauma generated during CI insertion and the resulting tissue trauma and chronic inflammatory response can damage residual acoustic hearing. Residual acoustic hearing can be beneficial when combined with CI electrical hearing (electro-acoustic hearing), and any interventions to preserve this would increase eligibility for CIs [37, 64, 66, 136]. Current means for detailed physical examination of the electrode-cochlear interactions involve animal [26, 86, 156] and human cadaveric testing [32, 72, 104]. However, these models present significant challenges as they cannot be easily instrumented or modified in shape and size to allow systematic testing of performance parameters. Furthermore, animal cochlea are very different anatomically from human cochleae [123] and also have restrictions on the availability and ethical considerations in their use. Only limited progress has been made towards other models, such as engineering realistic artificial cochleae [88].

A bio-mimetic cochlea has the potential to accelerate both the development of new treatments for hearing loss as well as optimise existing treatments. We evaluate requirements for a realistic model and assess current attempts to engineer artificial cochleae, their limitations, and indicate future directions for their development.

**Fig. 1:**
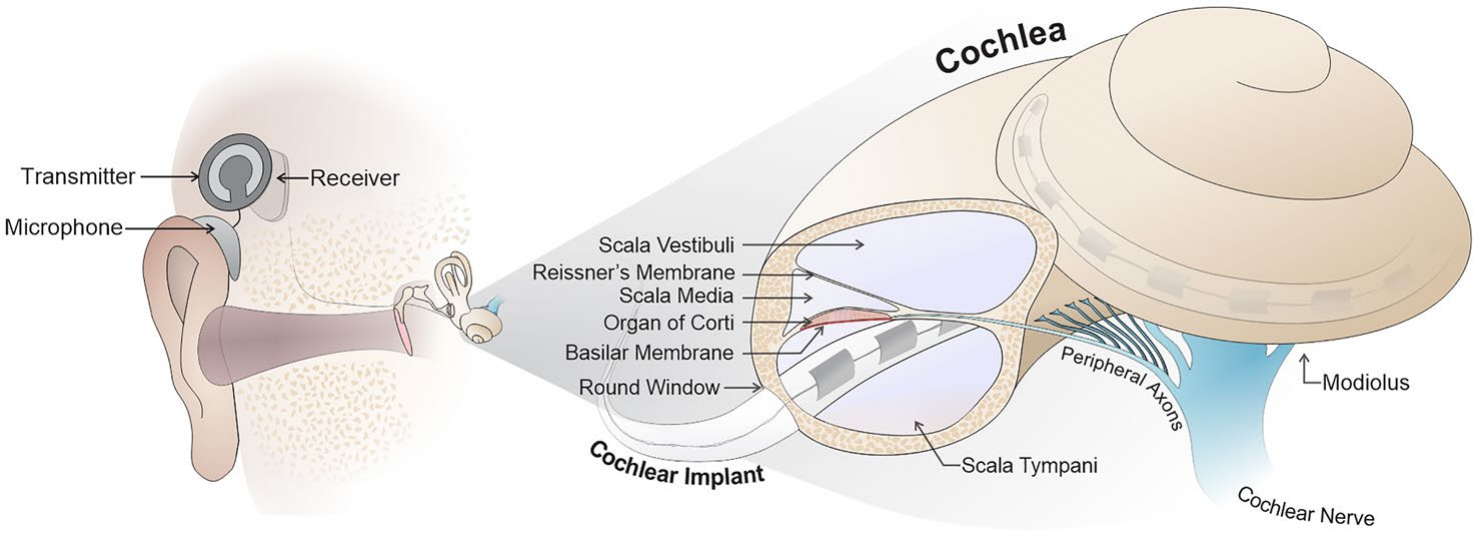
Diagram depicting the structure of the cochlea (right) and the insertion of the CI through the round window into the *scala tympani*. This is shown in relation to its position relative to the middle ear and external components of the CI (left). Note that spiral ganglion neurons from the cochlear nerve spiral around the 2.75 turns of the cochlea around the central axis (the modiolus)

## Biological Background of Cochlea

### Anatomy

Embedded in the temporal bone, the cochlea is a fluid-filled structure that is part of the osseous (bony) labyrinth, also referred to as the otic capsule. This consists of the semi-circular canals, responsible for sensing head rotation which is essential for balance, the vestibule, which houses the linear acceleration detectors (the otolith organs), and the cochlea itself (see Fig. 1).

Neural representation of frequencies in the normal cochlea are structured in a tonotopic manner, primarily by the intrinsic passive and active tuning of the basilar membrane, which maximises vibrations for particular frequencies in a graded manner from apex to base. This results in neurons (specifically spiral ganglion neurons localised in the Rosenthal’s canal) along the length of the cochlear spiral encoding low frequencies at the apex and high frequencies towards the base (in a range from 20 Hz to 20 kHz) [47]. CIs try to somewhat replicate this tonotopic representation, by presenting lower frequency signals to the apical electrodes and higher frequency ones to the basal electrodes.

A defining feature of the cochlea is its distinct ascending spiral geometry. One reason for the nautilus shell-like structure is thought to be due to spatial constraints in the temporal bone [113]. However, more recent studies have presented evidence suggesting that it also provides functional benefits. For instance, a study by Manoussaki et al. indicates that the graded curvature of the cochlea can aid the propagation of low frequencies similar to “whispering gallery nodes” and therefore influence low-frequency hearing limits [96].

**Fig. 2:**
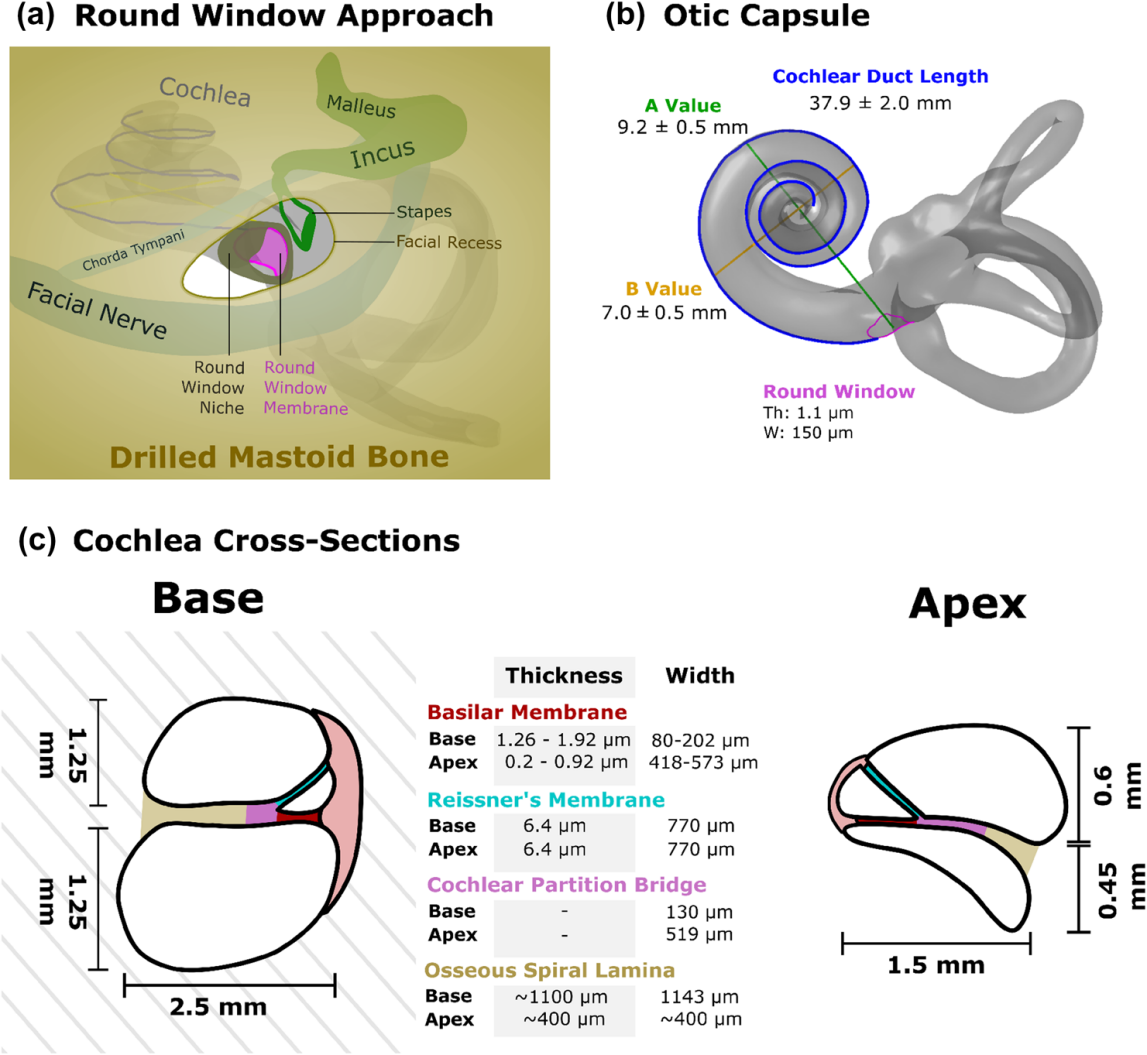
Limited surgical view of the round window during implantation (**a**) and Otic Capsule with cochlear duct length measurement (**b**). Cross-section of the cochlea at base and apex highlighting key anatomical features (**c**).* Th* thickness, *W* width

Key parts of the cochlea for surgeons are the bony round window (RW) niche and the round window itself, as they are the most common entry portal for CI insertion (see Fig. 2a). The RW niche is a bony pouch of the tympanic cavity located anterior to the RW which is closed with a membrane. The niche has a width and depth of about $1.66\pm 0.34$ mm and $1.34\pm 0.25$ mm, respectively [137]. The RW is a small, circular opening with a transverse diameter of about $1.65\pm 0.21$ mm positioned inferior and slightly posterior to the oval window at an average distance of 2–2.2 mm. It is covered by a thin membrane $(69.4\pm 4.3 {\upmu}\hbox {m}$) [126] called the round window membrane (RWM), which enables fluid movement within the cochlea during auditory stimulation [68, 133, 137]. The oval window is closed by the stapes footplate, a part of one of the three ossicles in the middle ear which transfer vibrations of the eardrum to the inner ear fluids. The oval window is set in the bony vestibule. During auditory stimulation, the stapes footplate vibrates, creating inner ear pressure which is released by the compliant RW membrane.

### Variation in Cochlear Anatomy

Human cochleae display large variations in both size and shape [9, 140], which likely affects the clinical performance of CIs. It is, therefore, crucial to understand this variation when attempting to produce a representative artificial model. Indeed, by developing physical (i.e. model made of plastic or similar material that represents anatomically accurate “mechanical" twin of the human cochlea) or computational models that can represent the variation present in the human cochlea, we can try to understand the relationship between different structural features of the cochlea and the effectiveness of CIs. This could reveal possibilities for personalised/stratified medicine within this field by relating different device designs as being optimal for certain types of cochleae, particularly concerning the length of the cochlea, but also to other material properties such as stiffness of the implant in different dimensions for optimal, minimally traumatic full insertion.

Additionally, through a deeper comprehension of these disparities, researchers can enhance the utilisation of animal models by acknowledging the anatomical variations between the human and animal cochlea.

#### Characterisation of the Cochlea Size and Shape

The cochlea has a characteristic spiral geometry which flares out from a typical Archimedean spiral at the base. This has been characterised by either a piecewise function [24, 157], which separately describes the base and apex or a continuous double exponential function [16, 61]. Additionally, a function to determine the height of the cochlea is highly dependent on the reference frame used. For instance, when using a mid-modiolar axis, it is possible to determine that the height of the cochlear centerline increases linearly [24]. This can lead to the observation of three cochlea shape categories: sloping, intermediate, and ‘rollercoaster’ [9]. A sloping shape has an upward trajectory without any significant downward trend. The intermediate shape follows a slight upward trajectory after the RW, which then follows a slight decrease. Lastly, the ‘rollercoaster’ shape follows a downward trajectory after the RW, followed by an upward trajectory around 75–120°. Nevertheless, Gee et al. argue that by defining a basal plane sets the average height of the first 270° of the basal turn equivalent to the perspective of the inserted cochlear implant (CI) then the “rollercoaster” trajectory is not observed [43]. This definition results in a more sigmoidal increase of the cochlear height where the basal turn is rather flat, followed by a sharp defined rising of the cochlear spiral [43].

#### Size and Shape Variation of Cochlea

The variability of the cochlea is reflected in the range of cochlear duct lengths ranging from 30.8 to 43.2 mm [83, 155] and also, to a lesser extent, with the variable number of turns [9, 13, 49]. Hence, individual cochleae are not only a scaled-up version of the same basic shape but represent true morphologic variations. Therefore, different shapes and lengths of the electrode arrays should ideally be considered if atraumatic insertion, place-pitch matching (aligning the sound frequencies assigned to electrodes to the natural biologic tonotopic map), and preservation of residual hearing are of interest. Table 1 summarises some of the key anatomical features of the cochlea.

Furthermore, the height of the centre part of the *scala tympani* (ST) is larger than in the lateral and modiolar regions. The ST is the lumen into which CIs are placed [9]. Lateral wall height significantly decreases following the second turn (after 450°) [9, 74], which can imply a higher possibility of CI translocation through the basilar membrane from the ST (intended site) into the *scala vestibuli* or pushing against the basilar membrane. This likely increases the probability of residual hearing loss and perhaps also worse CI hearing outcomes, if the trauma affects the auditory neurons. Figure 2c depicts the changes in the cross-section of the cochlea and the width and thickness of its key membranes at the base and apex. The basilar membrane (excluding Organ of Corti) becomes thinner and wider towards the apex of the cochlea, ranging from 1.26 to 1.92 μm, thickness and ~ 202 μm width to 0.53–0.89 and 475 μm, respectively [99]. However, the thickness and width of the Reissner’s membrane remain consistent throughout the cochlear duct length at about $6.4\pm 2.6$ μm and $770\pm 180$ μm, respectively [31].

The basal turn represents a large part of the cochlear duct length. Its size variability is, therefore, a significant contributor to the overall CI insertion path and may drastically impact the angular insertion depth of the implant [38, 133]. Furthermore, the majority of insertion trauma is observable within this region (180–270°  from the RW) [1, 8, 33] which underlines its importance.

In addition to the major axis change between the first and ascending portions of the basal turn, several smaller anatomical peaks, dips and vertical jumps have been described in the vertical trajectory of the ST [9, 113]. These relatively sudden changes in the vertical trajectory of the ST can be critical when calculating the required implant insertion depth in 3D. Therefore, it is essential to replicate them in detail in the physical or computational artificial model as they may play a significant role in determining the insertion interactions between the cochlea and the implant. Each cochlear shape category is unique and, in theory, could require slightly different approaches in terms of insertion site and angle to minimise trauma. However, recent evidence shows a relative independence of the overall friction force experienced by the implant to the cochlea shape [61], although shape may still play a role in the local stresses and trajectories of the implant, and where this force is concentrated.

#### Variations of Round Window

The RW also demonstrates large variability in shapes between implant recipients with majority being of oval-shape (see Table 1) [29, 95, 130, 132]. A sharp bony crest called the *crista fenestra*, which occupies an extensive area projecting into the RW, may play a significant role as a barrier to the ST [29]. Different RW niche morphologies produce various sizes of crests [6]. In some situations, removing the crest is necessary to introduce the implant successfully. If the RW is unreachable or the entry angle is not satisfactory, a cochleostomy (separate hole drilled into the ST) is typically considered. Additionally, the surgical view of the RW is limited by the margins of facial recess, i.e. facial nerve posterior, *chorda tympani*, eardrum anterior, and incus buttress superiorly (see Fig. 2a). Therefore, the surgeon’s manoeuvrability is restricted and could benefit from the experience acquired by training on a physical phantom of the human cochlea.

**Table 1:** Summary of the size and shape of various features of human cochleae

Component of cochlea	Measure			*N*	References
	Mean ± SD (range) (mm)				
Cochlear duct length	37.9 ± 2.0 (30.8–43.2)			436	[155]
	35.8 ± 2.0 (30.7–42.2)			310	[101]
	40.9 ± 2.0 mm			108	[113]
	Angular length 966.7° ± 45.1° (outer wall)				
	Number of turns 2.69 ± 0.13				
Number of turns	Number of turns		Percentage (%)		
	2.5		1	68	[13, 49]
	2.5		13		
	2.5–2.75		74		
	2.75–3		12		
Round window membrane	Height (mm)		Width (mm)		
	1.91 ± 0.78		1.37 ± 0.43	20	[130]
	0.69 ± 0.25		1.16 ± 0.47	34	[68]
	1.62 ± 0.77		1.15 ± 0.39	50	[132]
	Transverse diameter (mm)				
	1.65 ± 0.21			558	[137]
	Thickness (μm)				
	69.4 ± 4.3			37	[126]
Round window shape	Oval (60%), round (25%), and triangular (15%)			20	[130]
	Oval (50%), round (20%), triangular (12%), comma (10%), quadrangular (6%), and pear-shaped (2%)			50	[132]
Round window niche	Width (mm)		Depth (mm)		
	1.66 ± 0.34		1.34 ± 0.26	541, 460	[137]
Facial recess	Width (mm)				
	4.01 ± 0.56			356	[137]
Basilar membrane (excluding Organ of Corti)	Base		Apex		
	Width (μm)				
	~ 80		498	25	[152]
	138		573	Up to 15	[120]
	126		418	1	[90]
	201.9		475.2	Up to 14	[99]
	Thickness (μm)				
	1.26–1.92		0.53–0.89	Up to 13	[99]
	1.46–1.51		0.2–0.96	1	[90]
Organ of Corti	Base		Apex	Up to 15	[99]
	Height (μm)				
	66.02		62.69		
Reissner’s membrane	Width (spiral ligament to spiral limbus) (μm)			18	[31]
	770 ± 180				
	Thickness (μm)				
	6.4 ± 2.6				
Cochlear partition bridge	Base		Apex		
	Width (μm)				
	130		519	Up to 15	[120]
	228.6		499.2	Up to 13	[99]
Osseous spiral lamina	Base		Apex		
	Width (μm)				
	1143		~ 400	Up to 15	[120]
	726.6		335.9	Up to 13	[99]
	Thickness (μm)				
	1100		~ 400	Up to 15	[120]
Helicotrema	Length (along lateral wall) (mm)			14	[53]
	1.6 ± 0.9				
Scala tympani		Width (mm)	Height (mm)		
	Base (0°)	2.5 ± 0.3	0.9 ± 0.25	9	[34]
	Apex (900°)	1.2 ± 0.3	0.4 ± 0.1		
Scala vestibuli		Width (mm)	Height (mm)		
	Base (0°)	2.5 ± 0.2	1.3 ± 0.1	9	[34]
	Apex (900°)	1.3 ± 0.3	0.5 ± 0.15		

*SD* standard deviation, *N* number of samples

## Reproduction of Cochleae

Models of the cochlea can be broadly classified into four categories: physical models, animal models, tissue engineering models, and computational models. Physical models are useful for investigating the mechanical aspects of CI insertion and the electrical properties of the electrode-nerve interface [74, 88, 105]. These models are often created using materials that mimic the mechanical and electrical properties of the cochlea and can be modified to study the influence of specific parameters on CI behaviour. Animal models, such as guinea pigs [4, 51, 58] and chinchillas [145], are prevalent in CI research due to their ability to replicate physiological responses to stimulation, or damage. Tissue engineered models are created using living cells and could be potentially used to study the effects of CIs on cells and tissues in more biologically realistic environments than 2D cultures on tissue culture plastic. In general, tissue-engineered models are often constructed using scaffolds that support the growth and organisation of cells into tissues. For cochleae, there have been examples of organoid cultures [84] and decellularised tissues [100] for cochlear tissue engineering although more extensive studies of producing replacement tissues have been discussed for the middle and outer ear [3, 5, 22]. Lastly, computational models are increasingly being used to study the electrical and mechanical properties of the cochlea and the effects of CIs on the auditory system [7, 14, 110, 118]. These models can be used to replicate the complex biological environment of the cochlea in a more controlled and reproducible manner and one that can be easily modified to study specific parameters. Computational models could also be used to predict the performance of CIs in different scenarios, such as differing electrode configurations or stimulation strategies.

The aforementioned models often work in tandem as the acquired data from physical, animal, and tissue-engineered models can be subsequently fed into computational simulation models to accelerate research and examine variables that would otherwise be substantially time-consuming to study. These models play an imperative role in validating computational simulations, as the simulations fail without their tangible data and confirmation.

The following sections discuss various techniques to produce physical artificial cochlea models and the utilisation of animal models, tissue engineering models, and computational simulations.

### Physical Models

The development of an anatomically accurate model of the human cochlea is of great interest to researchers studying the mechanical aspects of CI insertion (e.g. atraumatic implantation and insertion trajectories) and electrical properties in optimising the electrode-nerve interface (e.g. simulating nerve activation with different stimulation strategies and electrode positions). For example, having a model that can reliably measure insertion forces and register it with implant position over time (e.g. insertion depth) delivers information that can influence surgical practice. Studying the behaviour of electrode arrays within the cochlea can improve CI design and introduce an individualised approach by understanding how a specific implant might behave for a given recipient based on their cochlear shape and size, allowing a personalised selection of implants to possibly minimise insertion forces, optimised electrode position, or avoid basilar membrane contact.

The properties of the artificial cochlea model may vary based on the experiment. For instance, a transparent physical model with a smooth intracochlear lumen with embedded sensors is required to evaluate the intracochlear pressure or insertion forces [54, 102, 105, 124]. The transparency of the model is essential as it allows direct visualisation of the implant behaviour during insertion [89]. The insertion forces can be measured using a force sensor that can be attached to the cochlear model (often multi-axis measurement) [62, 89, 109], between the electrode array and the insertion device (one-axis) [82, 85]. Alternatively, in the case of an “open-channel" artificial model (a model with only the basal turn of the ST fully open at the top surface), the overall force at the location of the basilar membrane/ osseous spiral lamina can be measured with flat force sensor or membrane [54].

Nevertheless, in order to create these models, it is vital to understand the mechanical and electrical properties of the cochlea. Table 2 displays key mechanical data that should be considered when fabricating an artificial cochlea. For instance, the load required to rupture the basilar membrane, which is in the basal turn and apical turn about 35 and 26 mN [67], respectively, is of great interest when mimicking CI insertion as its penetration can result in the translocation of the CI into the *scala media* and *scala vestibuli*, which is frequently associated with the loss of residual hearing, and worse CI function [57, 74, 81, 111, 150, 154].

**Table 2:** Approximate values for the mechanical properties of different components of the cochlea

Component of cochlea	Rupture load (mN)	Young’s modulus (MPa)	Notes	References
Basilar membrane	Apical turn: 26	Apical turn: 6.4	58 years, woman	[67]
	Middle turn: 33	Middle turn: 6.0		
	Basal turn: 35	Basal turn: 9.7		
Round window membrane	564	9.8	69 years, man	[67]
		Storage modulus (*G*′): 2.32 to 3.83 Loss modulus (*G*′): 0.085 to 0.925		[158]
Reissner’s membrane	4.2	34.2	58 years, man	[67]
Osseus spiral lamina	44–122	–	10 cadaveric samples; OSL, BM, RM measured together	[129]

Note values were derived from measurements of the specific tissues where possible

* OSL* osseus spiral lamina, *BM* basilar membrane, *RM* Reissner’s membrane

Additionally, if we are able to replicate the electrical properties (e.g. resistivity) of the cochlear bone and its contained fluids (see Table 3), this would greatly help in understanding current spread during cochlear electric stimulation. Having artificial models can vary significantly increase the number of repeated experiments that can be performed in a standardised model [61, 88], as opposed to biological tissues, and thus the robustness of any conclusions; these models are mechanically more durable than cadaveric specimens and do not degrade quickly over time. Furthermore, the use of fixed cadaveric tissues may alter the electrical and mechanical properties of the sample, and the availability of fresh cadavers is often limited. Artificial models can also be altered to change one parameter at a time in order to explore the influence of specific parameters. However, replicating the electrical properties of a cochlea has proven to be difficult. Artificial cochleae could be created from bone-like material to better mimic in vivo tissue; nevertheless, these materials are yet to be fully characterised for high-resolution additive manufacturing. One interesting solution is that 3D printing can be used to fabricate models with an appropriate size of embedded pores filled with a conductive solution that would enable fine-tuning the electrical properties of the material [28, 88, 106].

**Table 3:** Approximate values for the electrical properties of selected anatomical elements of the cochlea

Anatomical element	Electrical conductivity (Sm^−1^)	References
Perilymph	1.43*–1.78**	[12, 114]
Endolymph	1.68	[12]
Stria vascularis	0.0053	[14]
Basilar membrane	0.027–0.375	[14, 40, 149]
Reissner’s membrane	0.0006–0.00098	[14, 40]
Temporal bone	0.0156	[94, 115]
Spiral ligament	1.67	[14]

*Based on similar ionic composition of cerebrospinal fluid and **saline

**Fig. 3:**
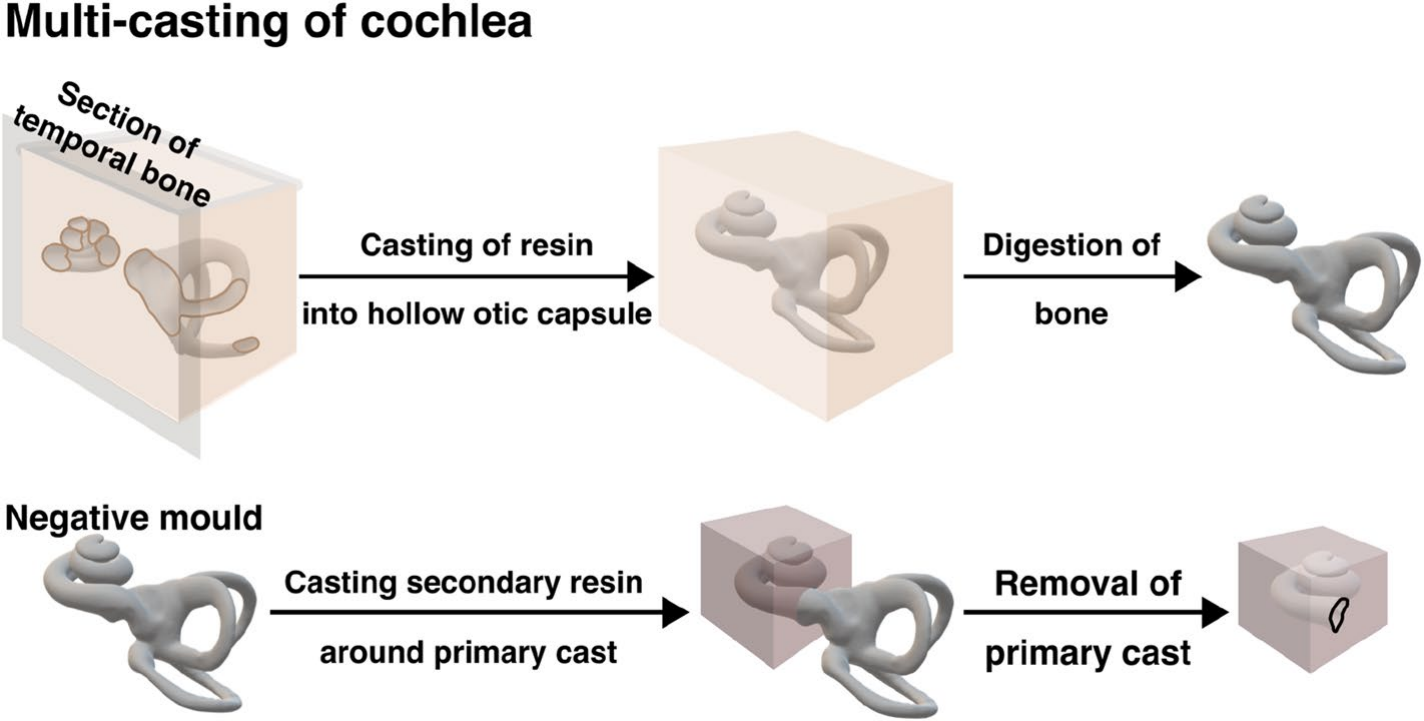
Workflow of corrosion casting method. This method uses curable resins that fill the hollow otic capsule within the temporal bone, which is digested to leave the cured resin that replicates the otic capsule space. Negative moulds utilise a double casting method, using the initial cast as a mould which is subsequently removed to leave the hollow lumen of the cochlea

**Fig. 4:**
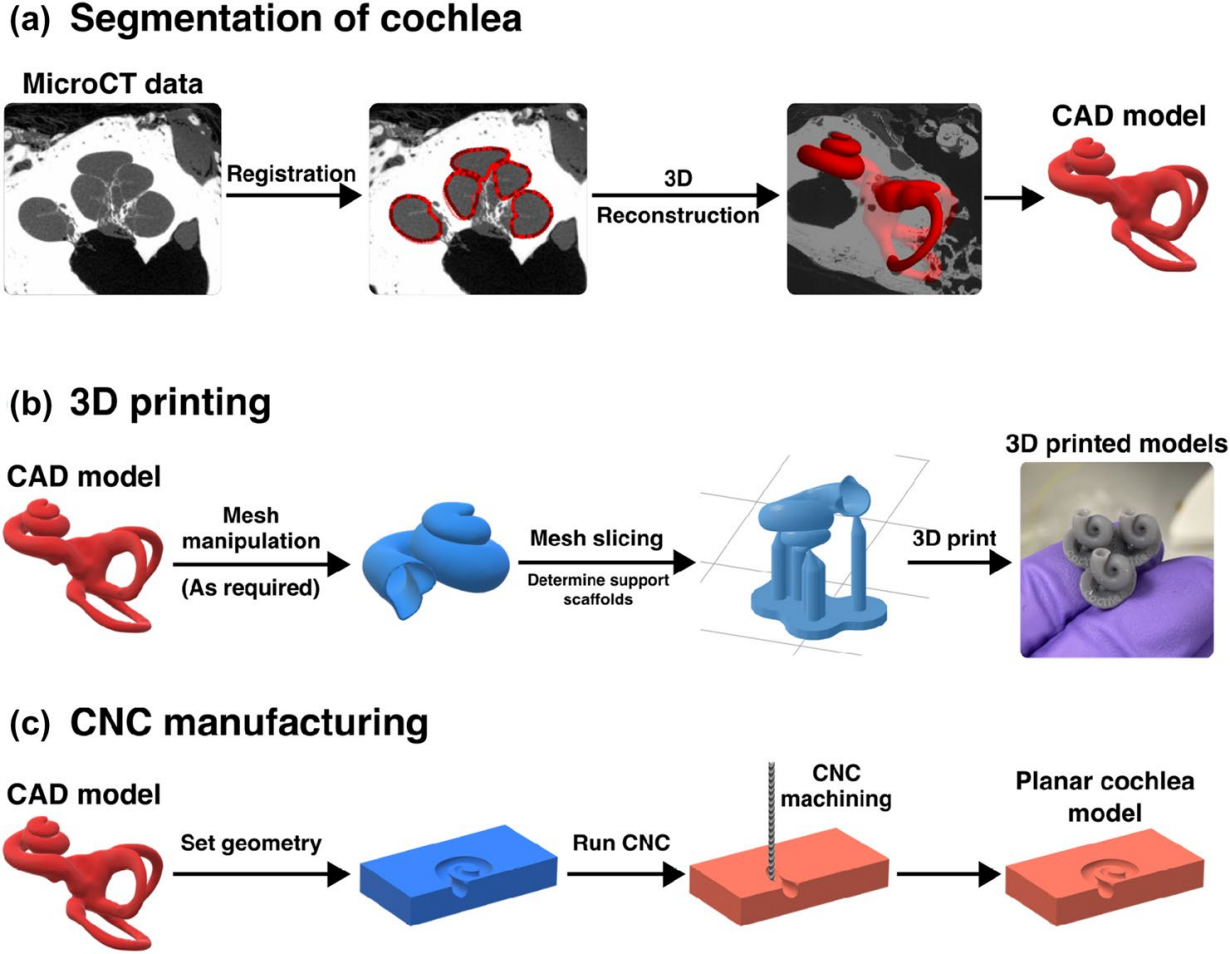
Workflow of 3D printing and CNC machining artificial cochlea. **a** Registration and segmentation of the otic capsule from microCT scans of the temporal bone can be used to generate CAD files of the cochlear structure. **b** CAD files can be manipulated to make various geometries for 3D printing. For resin-based 3D printing, scaffolding and slicing the model are required prior to printing shape-accurate models. **c** CNC—compatible files can be derived from cochlear CAD models that can be programmed to machine planar cochlear models

Several methods can be utilised for the development of artificial cochleae (see Figs. 3 and 4).

#### Casting

For a long time, casting has been the prevalent technique for developing artificial cochleae. Several studies have utilised polymethylmethacrylate (PMMA, often known as acrylic glass or plexiglass) as the casted material for the fabrication of 2D [109, 122] and 3D [18, 82, 89, 102, 103, 122] cochleae models. The advantage of this material is its transparency which provides good visualisation of the array’s behaviour during insertion. However, the 2D models offer only limited information as they lack the true three-dimensional form [24].

Rebscher et al. described a multi-casting process exploiting low-melting-point alloy (LMA) and PMMA for the development of an artificial ST (see Fig. 3) [122]. They produced multiple high-accuracy replicas of the same temporal bone by utilising vulcanising silicone rubber, which functioned as a mould. Firstly, the PMMA was injected into a dissected temporal bone and subsequently cooled down. To remove the ST cast from the cadaveric specimen, the temporal bone was decalcified. Following that, the PMMA cast was then covered by vulcanising silicone creating mould. After the curing process of the silicone, the mould was carefully divided into two parts to release the original PMMA cast. In the next step, LMA was poured into the silicone mould to create LMA-casted replicas. Lastly, LMA casts were covered by PMMA. Once PMMA cooled down, the LMA was released (lower melting point than PMMA) to fabricate a “block" model of the ST.

This process of multi-casting is relatively complicated and time-consuming, in addition to the work needed for the dissection of temporal bones from cadaveric specimens. Moreover, the number of fabricated models using Rebscher’s method is limited by the number of dissected samples, and the models’ shape cannot be easily modified or adjusted, as it is based on the anatomical specimen itself. Many studies have exploited casted cochlea models from companies such as Advanced Bionics (AB), MED-EL or Cochlear [24, 82, 89, 93]. However, dimensions and anatomic accuracy of these models can be sub-optimal compared to other manufacturing techniques, although this has not been systematically quantified yet [24, 89]. As multi-casting involves many steps, each inherently introducing a level of variability, it can be assumed that the anatomic accuracy will suffer as small features could be difficult to reproduce.

In addition to producing cochlear models, corrosion casting has been used to study various anatomical features of the cochlea. For example, Carraro et al. have used partial corrosion casting to study the vasculature within the cochlea [20, 21]. By perfusing the vasculature with a castable commercial resin, Mercox II, and digesting tissue, it was possible to preserve the vascular structure in mouse cochleae and study it with scanning electron microscopy.

#### Additive Manufacturing

Additive manufacturing, such as 3D printing, is now a well-proven technology that has the potential to develop highly accurate artificial models of human cochleae. Nevertheless, not all 3D printing techniques are suitable for developing such models. Firstly, some 3D printing technologies, such as fused deposition modelling (FDM) or selective laser sintering (SLS), do not produce products with the level of detail required for cochlear research. This is a result of the materials used as well as the nature of the technique. SLS exploits a laser to sinter plastic particles (often nylon-based material) into a solid structure. Although SLS does not require support generation during printing as the powder supports the print, its limitation is the printing resolution, which is suboptimal for prints of the inner ear very small size. FDM may utilise transparent materials (e.g. PLA); however, it fabricates products by heating a filament and building it layer-by-layer, which may generate a step-like finish with a low level of smoothness. The printing resolution is, therefore, significantly dependent on the layer’s height. This limitation may further result in suboptimal transparency of the product as each layer scatters light. Secondly, the cochlea anatomy is complex as it contains overhangs, tunnels, and hollow structures, and its fabrication may require temporary supportive scaffolds. If the supports are erected within the cochlea lumen, the smoothness of the inside structure may be compromised. Following that, after the removal of the supportive scaffold, the print is frequently polished to obtain better surface smoothness. However, due to the complex anatomy and inaccessibility; this is not easily achievable in the cochlea.

Some 3D printing techniques such as stereolithography (SLA, see Fig. 4b) [18, 39, 54, 89, 108], digital light processing (DLP) [61], and polyjet printing (PJP) [24, 89] have been already exploited for the fabrication of the ST models with much better accuracy (<40 ${\upmu }\hbox {m}$ [61]). SLA and DLP are photopolymer-based technologies that use ultraviolet light to cure resin (liquid plastics) into 3D prints. In the case of SLA, a system of mirrors focusses the laser into a small spot that is subsequently moved over the printing plane to cure each layer. A single accurate light source provides good smoothness as each printed layer is merged with the previous one. In addition, the layer-merging process also decreases the number of needed supports. DLP uses a projector with UV light to cure the whole layer at once, which enables faster printing but might produce a step-like finish with a too-high layer height. For SLA and DLP, the printed product must be further processed after the printing by washing in isopropanol and curing using a UV-light chamber to obtain the highest possible quality. The optimal printing quality of these techniques comes with a trade-off, as the only supported materials are photopolymer-based. Furthermore, SLA and DLP enable the printing of the product from one material only. However, for example, the material’s conductive properties might be tuned by introducing microchannels or pores for modifying the electrical conductivity of the construct, used when studying CI stimulation electrical properties [88].

PJP techniques, on the other hand, allow the printing of multiple materials at once and, thus, fabricating products with various mechanical properties (e.g. flexible and rigid). It exploits thermoplastics that are heated and then deposited on a platform layer-by-layer in the form of droplets using multiple print heads. Moreover, the supportive scaffolds can be printed out of soluble materials, which are easier to remove than SLA and DLP supports which are made of the same materials as the print.

Leon et al. used both PJP and SLA printing techniques to fabricate an artificial model of ST and compared them to models from CI companies MED-EL (SLA printed), AB (multi-step casting) and Cochlear (2D planar model) [89]. They observed that the measured insertion forces in the SLA model were similar to Cochlear’s model, which demonstrates good inner surface smoothness. However, the PJP model demonstrated even lower insertion forces than the SLA printed model, implying an improved internal surface finish. The disadvantage of the PJP model was its semi-transparency which was not optimal for successful visualisation of the implant behaviour during insertion. Hence, they recommend the SLA technique due to its ability to fabricate transparent models. However, PJP technique can achieve enhanced transparency with the use of appropriate material, for example, VeroClear (Stratasys) [112].

It is important to bear in mind that 3D printing capabilities progress rapidly. Several new materials are developed each year, enhancing 3D printing abilities and providing new avenues for prototyping [23, 116]. Hence, different 3D printing techniques can be recommended for the fabrication of artificial cochlea each year as the material’s selection vary.

#### Other Manufacturing Methods

Some studies have used 2D polytetrafluorethylene (PTFE) artificial cochleae [85, 124]. This material was used due to its low friction coefficient, which was found to be comparable to the slippery endosteum of the ST [79, 144]. These models can be prepared by using computer numerical control (CNC, see Fig. 4c) machines that carve the material in a 2D plane using precise drills. Although the PTFE material provides optimal smoothness of the internal surface of the model, the lack of the third dimension significantly alters the electrode array’s behaviour during insertion [24, 89].

#### Electro-Acoustic Models

As there are many aspects of cochlear physiology that are of interest to researchers, there are a variety of different models to replicate these different aspects. In addition to the physical mentioned above, some have investigated modelling electro-acoustic aspects of the cochlea. These typically attempt to replicate the sensory epithelium of the cochlea using electro-active materials such as piezoelectric membranes and micromechanical systems [63, 65, 70, 146, 159]. Replicating the high-frequency selectivity (20 Hz–20 kHz) and sensitivity, sound pressure level range (0–140 dB SPL) as well as the small size and power requirements of the human cochlea, presents a substantial technical challenge; however, work continues towards the aim of restoring the range and specificity of natural hearing with CIs [19, 107]. This may involve atraumatic insertion covering the full extent of cochlear spiral and an increasing specificity of the neural activation. Currently, devices have demonstrated some limited tonotopy within the range of human speech, typically in the ~ 1.4 to 14 kHz range with rather high 70 dB+ sound pressure levels [70, 159]. Other reports have used alternative methods such as triboelectric devices to detect lower frequency ranges from ~ 300 to 2000 Hz in in vivo conditions with Guinea pigs [69] with idealised conditions in other studies lowering the minimum frequency to the tens of hertz [91].

### Animal Models

Animal models are well established in CI research and have benefits as models in replicating the complex structure of the cochlea and its constituent tissues. By conducting in vivo experiments, it is possible to use the features of intact myelinated primary auditory neurons/ spiral ganglion neurons within the cochlea, an immunological response which is important in understanding chronic issues such as fibrosis and ossification [25, 26, 131], and potential for conducting some behavioural studies and measuring electrically evoked potentials [35, 46, 50, 80] to conduct a wide range of CI studies to understand the CI-nerve interface.

There are, however, several considerations in the applicability of different animal models for human CI research in terms of both their anatomy and physiology. The cochlea varies in size, shape, and complexity amongst different species, with differences in the length, width, and number of turns [73, 78, 123]. Although the overall scalar structure is largely conserved in mammals, the overall shape and size do not scale linearly with overall body size, indicating that factors other than body size determine the cochlea’s structure [78]. As most animals would have much smaller cochleae than humans, smaller custom-made small CIs will need to be used, which increases the complexity of conducting and extrapolating results from animal models [86, 92, 121]. Furthermore, the structure of the cochlea will determine the spread of the electric field from a CI and, hence, the neural activation which limits their effectiveness in answering some of the key unanswered questions in the CI field such as the spread of neural activation to different CI parameters.

Several different animal models have been used for studies that are interested in structure-related parameters and CI implantation. Rodent models such as mice, rats, gerbils, guinea pigs, ferrets, and chinchillas have been widely used in CI research due to their availability, potential for instrumentation, and established gene-editing tools in the case of mice and rats; as reviewed by Reiss [123]. Guinea pigs, in particular, have been extensively used due to their wide availability, their inner ears being easy to access, and their cochleae being more comparable to the human cochlea in several physiological aspects [4, 51, 58, 86, 147]. Chinchillas have been considered an even better model due to the similarities of their cochlea to humans with regards to its number of turns, hearing range, and sensitivity [145]. Cats are the most popular non-rodent model due to their basal turn being of a similar size to human cochlear [123], although, the overall shape and size of their cochleae differ significantly from humans. Moreover, the lack of myelin in the soma regions of human type I primary auditory neurons causes a delay in spike conduction compared to cat neurons [119], which can impact the transmission of temporal fine structure of auditory signals in the human cochlea. Larger animal models include sheep [128] and miniature pigs [156] as well as marmoset [73] and rhesus macaque monkeys [97, 123]. These are closer to the size of human cochleae, with the marmoset cochlear shape being most similar when scaled up by a factor of 2.5 [73], and allow the partial implantation of clinical electrodes although these model are much less available than small rodents and do not well replicate many of the specific features of human cochleae which are not even featured in other primates [151]. For comparison, the ratio of the volume of the human cochlea to different animal cochleae is as follows: mouse 80–100:1, rat 20–25:1, gerbil 5–6:1, cat 2–3:1, macaque 2–3:1, and sheep ~ 1.7:1 [123].

It is important to consider that the use of animal models is complementary to measures that can be conducted in human patients and cadaveric tissues. For instance, several studies have characterised the electrical properties [32, 72] and conducted CI insertion studies [18, 75, 77] in both fresh-frozen and fixed human cochleae. Furthermore, many electrophysiological and psychoacoustic measures have been developed to test the CI-nerve interface in humans, which include contact impedance and trans-impedance measurements [59, 87, 138], electrically evoked compound action potentials (eCAPS) [27, 41, 42], and electrically evoked auditory brainstem responses (eABR) [17, 36] which enable the evaluation of CI electrical characteristics (including fibrosis, positioning, and electrical faults), cochlear neural activation patterns, and propagation the CI stimulation to the brain, respectively. However, human studies do not allow us to systematically test numerous parameters in the same experiment, such as electrode position, stimulation parameters, pulse shapes, the geometry of the cochlea, and electrode design, size, and shape, which are much more possible in other models.

In conclusion, animal models are valuable in conducting validation of CI techniques and the systemic responses to CI implantation. However, the limitations of these different models should be considered, especially in light of reducing animal use and used in conjunction with human measures and other models, as will be explored below.

### Tissue Engineering

Several animal models have been utilised to study the cochlea and develop strategies to improve CI performance. Yet, it remains unclear how to improve electrical stimulation and how different stimulation strategies could affect neural excitation. This necessity led to the use of tissue engineering in the hearing field.

Tissue engineering is a set of methods that can replace or repair damaged or diseased tissues with natural, synthetic, or semi-synthetic tissues which can be fully functional or will grow into the required functionality [143]. These methods could, in theory, be utilised for replicating the complex three-dimensional cellular architecture of the cochlea in vitro. Furthermore, they could serve as useful platforms for studying cellular viability and expression in various conditions.

Two important cell types in the cochlea are hair cells (HCs) and primary auditory neurons (PANs), also known as spiral ganglion cells (SGNs). In the mammalian cochlea, HCs serve to sense the mechanical movement, amplify it and transmit this signal to the auditory nerve [44, 135]. PANs act as the neural conduit transmitting cochlear HC signals to the brain [30].

Some research has focussed on culturing auditory cells obtained from animals or differentiated from induced pluripotent stem cells (iPSCs). Much work has been conducted in vitro, which mostly use mouse, rat, and guinea pig sources for auditory HCs and PANs. Although it has been quite challenging to obtain these cells in significant numbers and maintain them over time, especially when requiring specific purified cell populations, recent studies have demonstrated some success [98, 117]. A few studies have progressed to generating human inner ear cells (e.g. IHCs and OHCs) from iPSCs [45, 60, 71]. It has been more challenging to differentiate PANs since the concerns would involve the electrical activity, firing potentials, and the possession of appropriate ion channels as well as the gene expression profiles.

Whilst in vitro models have facilitated the understanding of cellular mechanisms within the cochlea, they are limited in replicating the complexity of the in vivo micro-environment.

By combining data from the flexibility and specificity of in vitro experiments, systematic effects and replication of live structures of in vivo studies, and the clinical relevance of cadaveric studies, much has been learned about the cochlea and the impact of CIs. However, as all of these approaches have their limitations, there is an unmet need for in vitro platforms for hearing research. The cellular and molecular aspects of the cochlea could be integrated into a 3D model, which would complement the limitations of the previous models. This in vitro platform could mimic the main functional aspects of the cochlea, including the current spread profile. If incorporated as a host to human iPSC-derived cells, this model would not only reduce the time and cost required for testing but also eliminate the need for experiments on living creatures to study cochlear biology and determine the efficiency and reliability of new drugs or technologies, e.g. CIs, for hearing research.

### Computational Models

There is a large field of computational audiology that has been used to effectively model several aspects of the cochlea which we will briefly overview in this review. In terms of modelling physical aspects of the cochlea, these can broadly be categorised as electrical and mechanical models.

Electrical models of the cochlea are focussed on optimising the electrical implant-nerve interfaces that underlie the function of a CI and have been reviewed extensively by others [2, 48, 76]. These models primarily consist of two main aspects: (1) modelling of the electrical voltage spread within the cochlea, and (2) biophysical and phenomenological models of the neural excitation of auditory nerve fibres.

For the 3D electrical characteristics of the cochlea, there has been extensive work in developing finite element models of the electrical stimulation of CIs that have been established by the groups of Frijns and Rattay [14, 118]. These have gradually increased in complexity from simpler parametric representations of the cochlear spiral to microCT-based models that also incorporate the trajectories of auditory neurons [11, 55, 115]. As well as understanding the electrical properties of the cochlea, these finite element models have also been utilised for impedance-guided insertion to determine the CI positioning within the cochlea from electrical measurements [127]. These finite element models can be coupled to multi-physics simulations such as thermal safety analyses of intracochlear heating with magnetically steered CIs [39]. As an alternative to finite element models, simpler circuit models of the cochlea have been developed, such as ladder network models, to model specific phenomena [148].

Biophysical models of neural activation are extensions of the foundational work of Hodgkin and Huxley [56]. As discussed by Bachmaier [10], the use of multi-compartmental biophysical models of myelinated nerve fibres is able to replicate many phenomena observed in patients such as the sensitivity of the auditory nerves to the polarity of stimulation [15, 118, 134].

In contrast to the biophysical approach, phenomenological models do not rely on specific biophysical mechanisms and derive empirical relationships based on neurophysiological and psychophysical observations [141]. Due to the much-reduced parameter space, this approach allows the efficient modelling of complex phenomena that can be adjusted to individual CI patients and has proven effective at predicting and explaining a diverse range of auditory phenomena [139, 141, 142].

Combining the 3D volume conduction models with neuronal models can be a compelling method to investigate the effect of various parameters of the electrode-nerve interface for CIs. These enable the investigation of the effect of different stimulation parameters and positioning on auditory nerve fibre activation [11, 55, 94, 115]. Recent studies have demonstrated the coupling of neural activation from these models to an automatic speech recognition neural network to predict phoneme-level speech perception and information transmission [16].

The mechanics of the cochlea have been extensively studied since the pioneering work of von Békésy [149]. The extensive work in the mathematical and computational modelling of the basilar membrane micromechanics that underlie the mechanism of acoustic hearing is reviewed by Ni and colleagues [110]. Finally, mechanical models can provide insight into the insertion forces during cochlear implantation, which can lead to significant trauma and inflammatory response, damaging residual hearing [7].

In conclusion, computational models can be powerful tools to facilitate the understanding of the physical phenomena within the cochlea. However, these models require the correct inputs for measured quantities that can often be difficult to derive and need validation with experimental data to ensure that the model is accurate.

Tables 1, 2, and 3 summarise some of the essential information regarding cochlea physical, electrical, and mechanical attributes that can be used for computational simulations. Table 4 compares the merits and drawbacks of the examined cochlea models.

**Table 4:** Advantages and disadvantages of discussed types of cochlea models

	Advantages	Disadvantages
Physical models	Systematically modifiable and reproducible	Limited trauma prediction
Animal models	Suitability for testing physiological and inflammatory responses in vivo	Difference in cochlear anatomy and physiology
Tissue engineering	Possibility of manipulation and reproducibility	Limited replication of human cochlear microenvironment
Computational models	Systematic modification, flexibility, integration of multiple models	Requires validation and accurate parameterization, difficult to model complex non-linear behaviours

## Future Perspectives

Future research will focus on developing anatomically accurate artificial cochlea with embedded force and pressure sensors to detect insertion forces that arise during CI implantation. Preserving the residual hearing will aid the further development of EAS implants as natural acoustic stimulation is yet to be exceeded in performance by the electrical stimulation. Additionally, these models could also be utilised for studying inner ear therapeutics and drug delivery systems. Accurate, transparent cochlea models could help precisely determine the pharmacokinetics of drugs delivered inside the inner ear and their spread over time.

Combining cell-based models with animal models can lead to a more comprehensive understanding of CIs and improve the design of safe and effective treatments for auditory disorders. Cell-based models can be used to simulate the electrical and mechanical properties of the cochlea and to study how different stimulation parameters affect the auditory nerve. This information can then be used to guide the design of animal experiments, such as determining the optimal stimulation parameters to use in vivo. Animal models can be used to validate and refine the cell-based models and verify their accuracy in replicating the in vivo response of the cochlea to CIs. A cell-based 3D model of the cochlea could also play an important role in understanding the pathophysiology and aetiology of auditory disorders as well as allowing the interpretation of electric fields of the electrode arrays of CIs in the cochlea by bio-mimicking the true cochlear physiology. Despite the limitations of animal models, they still have advantages in certain areas, such as tracking the systemic response to cochlear implantation and aiding in the development of new therapeutic approaches to mitigate potential adverse effects.

The data from in vivo and in vitro experiments enables us to validate and inform the design of computational models to understand the mechanisms of the CI-auditory nerve interface. These computational models have increased in complexity over the last 20 years in development to combine finite element models with auditory nerve models to test a variety of clinically relevant parameters and help devise new stimulation strategies. Further development in this field may enable personalised approaches to replicate an individual’s specific cochlear anatomy and CI interface to improve their performance rather than generic procedures. Additionally, the dynamic time-dependent component of CI stimulation, rather than purely resistive finite element models, could allow further insights into the validity of using specific stimulation parameters to improve focussed auditory nerve stimulation.

Ultimately, by combining the insights from patients, cadavers, animals, in vitro experiments, physical models, and computational models it is possible to account for their individual limitations and build a more comprehensive understanding of optimal CI application for patient benefit.

## Conclusion

The elaborate and intricate structure of the human auditory system is a marvel that cannot yet be matched by modern engineering. However, understanding the cochlear structure and how to interact with the delicate system is crucial in addressing huge challenges in otology and audiology. Improving models and understanding the cochlea will lay the foundation for developing the next generation of CIs and future inner ear therapies. These implants and treatments should address the major challenges of insertion trauma and current spread to preserve cochlear health and residual hearing whilst conveying high sound fidelity by improving the spatial selectivity of stimulation.

Furthermore, understanding the variability of cochlea’s anatomy and its effect on insertion parameters and CI performance could open up the capability of personalised approaches for individual cases to deliver optimal patient outcomes. Addressing these challenges will widen the eligibility for CIs and improve the lives of the growing proportion of people suffering from hearing loss.

Ultimately, a 3D in vitro model of the cochlea with integrated auditory cells would revolutionise the study of various features of the inner ear to support the development of new technologies and the validation of computational simulations and drug-based therapies.

## References

[1] Aebischer P, Mantokoudis G, Weder S, Anschuetz L, Caversaccio M, Wimmer W (2022). In-vitro study of speed and alignment angle in cochlear implant electrode array insertions. IEEE Transactions on Biomedical Engineering.

[2] Agrawal V, Newbold C (2012). Computer modelling of the cochlea and the cochlear implant: a review. Cochlear Implants International.

[3] Al-Qurayshi, Z., E. I. Wafa, M. K. Rossi Meyer, S. Owen, and A. K. Salem. Tissue engineering the pinna: comparison and characterization of human decellularized auricular biological scaffolds. *ACS Appl. Bio Mater.* 4(9):7234–7242, 2021. 10.1021/acsabm.1c00766 PMC8456428 34568774

[4] Albuquerque AAS, Rossato M, de Oliveira JAA, Hyppolito MA (2009). Understanding the anatomy of ears from guinea pigs and rats and its use in basic otologic research. Brazilian Journal of Otorhinolaryngology.

[5] Aleemardani M, Bagher Z, Farhadi M, Chahsetareh H, Najafi R, Eftekhari B, Seifalian A (2021). Can tissue engineering bring hope to the development of human tympanic membrane?. Tissue Engineering Part B: Reviews.

[6] Angeli R, Lavinsky J, Setogutti E, Lavinsky L (2017). The crista fenestra and its impact on the surgical approach to the scala tympani during cochlear implantation. Audiology and Neurotology.

[7] Areias, B., M. P. L. Parente, F. Gentil, and R. M. Natal Jorge. Finite element modelling of the surgical procedure for placement of a straight electrode array: mechanical and clinical consequences. *J. Biomech* 129:110812, 2021. 10.1016/j.jbiomech.2021.110812 34688063

[8] Avci E, Nauwelaers T, Hamacher V, Kral A (2017). Three-dimensional force profile during cochlear implantation depends on individual geometry and insertion trauma. Ear and Hearing.

[9] Avci E, Nauwelaers T, Lenarz T, Hamacher V, Kral A (2014). Variations in microanatomy of the human cochlea: variations in microanatomy of the human cochlea. Journal of Comparative Neurology.

[10] Bachmaier, R., J. Encke, M. Obando-Leitón, W. Hemmert, and S. Bai. Comparison of multi-compartment cable models of human auditory nerve fibers. *Front. Neurosci.* 13:1173, 2019. 10.3389/fnins.2019.01173 PMC6848226 31749676

[11] Bai S, Encke J, Obando-Leitón M, Weiß R, Schäfer F, Eberharter J, Böhnke F, Hemmert W (2019). Electrical stimulation in the human cochlea: a computational study based on high-resolution micro-CT scans. Frontiers in Neuroscience.

[12] Baumann SB, Wozny DR, Kelly SK, Meno FM (1997). The electrical conductivity of human cerebrospinal fluid at body temperature. IEEE transactions on bio-medical engineering.

[13] Biedron S, Westhofen M, Ilgner J (2009). On the number of turns in human cochleae:. Otology Neurotology.

[14] Briaire JJ, Frijns JHM (2000). Field patterns in a 3d tapered spiral model of the electrically stimulated cochlea. Hearing Research.

[15] Briaire JJ, Frijns JH (2005). Unraveling the electrically evoked compound action potential. Hearing Research.

[16] Brochier T, Schlittenlacher J, Roberts I, Goehring T, Jiang C, Vickers D, Bance M (2022). From microphone to phoneme: an end-to-end computational neural model for predicting speech perception with cochlear implants. IEEE transactions on bio-medical engineering.

[17] Brown CJ, Hughes ML, Luk B, Abbas PJ, Wolaver A, Gervais J (2000). The relationship between EAP and EABR thresholds and levels used to program the nucleus 24 speech processor: data from adults. Ear and Hearing.

[18] Bruns TL, Riojas KE, Ropella DS, Cavilla MS, Petruska AJ, Freeman MH, Labadie RF, Abbott JJ, Webster RJ (2020). Magnetically steered robotic insertion of cochlear-implant electrode arrays: system integration and first-in-cadaver results. IEEE Robotics and Automation Letters.

[19] Calero D, Paul S, Gesing A, Alves F, Cordioli JA (2018). A technical review and evaluation of implantable sensors for hearing devices. BioMedical Engineering OnLine.

[20] Carraro M, Negandhi J, Kuthubutheen J, Propst EJ, Kus L, Lin VYW, Harrison RV (2013). Attenuating cardiac pulsations within the cochlea: structure and function of tortuous vessels feeding stria vascularis. ISRN Otolaryngology.

[21] Carraro M, Park AH, Harrison RV (2016). Partial corrosion casting to assess cochlear vasculature in mouse models of presbycusis and CMV infection. Hearing Research.

[22] Chang B, Cornett A, Nourmohammadi Z, Law J, Weld B, Crotts SJ, Hollister SJ, Lombaert IM, Zopf DA (2021). Hybrid 3d-printed ear tissue scaffold with autologous cartilage mitigates soft tissue complications. The Laryngoscope.

[23] Chen X, Chen G, Wang G, Zhu P, Gao C (2020). Recent progress on 3d-printed polylactic acid and its applications in bone repair. Advanced Engineering Materials.

[24] Clark JR, Warren FM, Abbott JJ (2011). A scalable model for human scala-tympani phantoms. Journal of Medical Devices.

[25] Claussen AD, Quevedo RV, Kirk JR, Higgins T, Mostaert B, Rahman MT, Oleson J, Hernandez R, Hirose K, Hansen MR (2022). Chronic cochlear implantation with and without electric stimulation in a mouse model induces robust cochlear influx of CX3CR1+/GFP macrophages. Hearing Research.

[26] Claussen AD, Quevedo RV, Mostaert B, Kirk JR, Dueck WF, Hansen MR (2019). A mouse model of cochlear implantation with chronic electric stimulation. PLOS ONE.

[27] Cohen LT, Richardson LM, Saunders E, Cowan RSC (2003). Spatial spread of neural excitation in cochlear implant recipients: comparison of improved ECAP method and psychophysical forward masking. Hearing Research.

[28] Cooperstein I, Layani M, Magdassi S (2015). 3d printing of porous structures by UV-curable o/w emulsion for fabrication of conductive objects. Journal of Materials Chemistry C.

[29] Cornwall HL, Marway PS, Bance M (2021). A micro-computed tomography study of round window anatomy and implications for atraumatic cochlear implant insertion. Otology Neurotology: Official Publication of the American Otological Society, American Neurotology Society and European Academy of Otology and Neurotology.

[30] Dabdoub A, Fritzsch B, Popper AN, Fay RR (2016). The Primary Auditory Neurons of the Mammalian Cochlea.

[31] De Fraissinette A, Felix H, Hoffmann V, Johnsson L-G, Gleeson MJ (1993). Human reissner's membrane in patients with age-related normal hearing and with sensorineural hearing loss. ORL.

[32] De Rijk SR, Tam YC, Carlyon RP, Bance ML (2020). Detection of extra-cochlear electrodes in cochlear implants with electric field imaging/transimpedance measurements: a human cadaver study. Ear and Hearing.

[33] De Seta D, Torres R, Russo FY, Ferrary E, Kazmitcheff G, Heymann D, Amiaud J, Sterkers O, Bernardeschi D, Nguyen Y (2017). Damage to inner ear structure during cochlear implantation: correlation between insertion force and radio-histological findings in temporal bone specimens. Hearing Research.

[34] Demarcy T, Vandersteen C, Guevara N, Raffaelli C, Gnansia D, Ayache N, Delingette H (2017). Automated analysis of human cochlea shape variability from segmented microCT images. Computerized Medical Imaging and Graphics.

[35] DeMason C, Choudhury B, Ahmad F, Fitzpatrick DC, Wang J, Buchman CA, Adunka OF (2012). Electrophysiological properties of cochlear implantation in the gerbil using a flexible array. Ear and hearing.

[36] Di Stadio, A., L. Dipietro, A. De Lucia, F. Trabalzini, G. Ricci, F. Martines, V. Pastore, and A. d. Volpe. E-ABR in patients with cochlear implant: a comparison between patients with malformed cochlea and normal cochlea. *J. Int. Adv. Otol.* 15(2):215–221, 2019. 10.5152/iao.2019.6251 PMC6750786 31418713

[37] Dorman MF, Gifford RH, Spahr AJ, McKarns SA (2008). The benefits of combining acoustic and electric stimulation for the recognition of speech, voice and melodies. Audiology Neuro-Otology.

[38] Escudé B, James C, Deguine O, Cochard N, Eter E, Fraysse B (2006). The size of the cochlea and predictions of insertion depth angles for cochlear implant electrodes. Audiology and Neurotology.

[39] Esmailie F, Francoeur M, Ameel T (2021). Experimental validation of a three-dimensional heat transfer model within the scala tympani with application to magnetic cochlear implant surgery. IEEE Transactions on Biomedical Engineering.

[40] Finley CC, Holden TA, Holden LK, Whiting BR, Chole RA, Neely GJ, Hullar TE, Skinner MW (2008). Role of electrode placement as a contributor to variability in cochlear implant outcomes. Otology Neurotology.

[41] Garcia, C., J. M. Deeks, T. Goehring, D. Borsetto, M. Bance, and R. P. Carlyon. SpeedCAP: an efficient method for estimating neural activation patterns using electrically evoked compound action-potentials in cochlear implant users. *Ear Hear.*, 2022. 10.1097/AUD.0000000000001305 10.1097/AUD.0000000000001305 PMC10097494 36477611

[42] Garcia C, Goehring T, Cosentino S, Turner RE, Deeks JM, Brochier T, Rughooputh T, Bance M, Carlyon RP (2021). The panoramic ECAP method: estimating patient-specific patterns of current spread and neural health in cochlear implant users. Journal of the Association for Research in Otolaryngology: JARO.

[43] Gee AH, Zhao Y, Treece GM, Bance ML (2021). Practicable assessment of cochlear size and shape from clinical CT images. Scientific Reports.

[44] Groves, A. K., and D. M. Fekete. New directions in cochlear development. In Understanding the Cochlea, edited by G. A. Manley, A. W. Gummer, A. N. Popper, and R. R. Fay, Springer Handbook of Auditory Research. Cham: Springer, 2017, pp. 33–73.

[45] Gunewardene N, Crombie D, Dottori M, Nayagam BA (2016). Innervation of cochlear hair cells by human induced pluripotent stem cell-derived neurons. In Vitro Stem Cells International.

[46] Guo W, Yi H, Ren L, Chen L, Zhao L, Sun W, Yang S-M (2015). The morphology and electrophysiology of the cochlea of the miniature pig. The Anatomical Record.

[47] Hallowell, D., and R. Silverman. Hearing and deafness, 4th ed. *J. Acoust. Soc. Am.* 65(3):867–867, 1979.

[48] Hanekom T, Hanekom JJ (2016). Three-dimensional models of cochlear implants: a review of their development and how they could support management and maintenance of cochlear implant performance. Network (Bristol, England).

[49] Hardy M (1938). The length of the organ of corti in man. American Journal of Anatomy.

[50] Hartley DEH, Vongpaisal T, Xu J, Shepherd RK, King AJ, Isaiah A (2010). Bilateral cochlear implantation in the ferret: a novel animal model for behavioral studies. Journal of Neuroscience Methods.

[51] Heffner HE, Heffner RS (2007). Hearing ranges of laboratory animals. Journal of the American Association for Laboratory Animal Science.

[52] Helbig S, Settevendemie C, Mack M, Baumann U, Helbig M, Stöver T (2011). Evaluation of an electrode prototype for atraumatic cochlear implantation in hearing preservation candidates: preliminary results from a temporal bone study. Otology Neurotology.

[53] Helpard L, Li H, Rask-Andersen H, Ladak HM, Agrawal SK (2020). Characterization of the human helicotrema: implications for cochlear duct length and frequency mapping. Journal of Otolaryngology -Head Neck Surgery.

[54] Hendricks, C. M., M. S. Cavilla, D. E. Usevitch, T. L. Bruns, K. E. Riojas, L. Leon, R. J. Webster, F. M. Warren, and J. J. Abbott. Magnetic steering of robotically inserted lateral-wall cochlear-implant electrode arrays reduces forces on the basilar membrane in vitro. *Otol. Neurotol.* 42(7):1022–1030, 2021. 10.1097/MAO.0000000000003129 PMC8282696 33859137

[55] Heshmat, A., S. Sajedi, L. Johnson Chacko, N. Fischer, A. Schrott-Fischer, and F. Rattay. Dendritic degeneration of human auditory nerve fibers and its impact on the spiking pattern under regular conditions and during cochlear implant stimulation. *Front. Neurosci.* 14:599868, 2020. 10.3389/fnins.2020.599868 PMC7710996 33328872

[56] Hodgkin AL, Huxley AF (1952). A quantitative description of membrane current and its application to conduction and excitation in nerve. The Journal of Physiology.

[57] Holden LK, Finley CC, Firszt JB, Holden TA, Brenner C, Potts LG, Gotter BD, Vanderhoof SS, Mispagel K, Heydebrand G, Skinner MW (2013). Factors affecting open-set word recognition in adults with cochlear implants. Ear and Hearing.

[58] Honeder, C., N. Ahmadi, A.-M. Kramer, C. Zhu, N. Saidov, and C. Arnoldner. Cochlear implantation in the guinea pig. J. Vis. Exp. (JoVE) (136):56829, 2018. 10.3791/56829 PMC6101746 29985368

[59] Hoppe, U., G. Brademann, T. Stöver, A. R. d. Miguel, R. Cowan, M. Manrique, J. C. Falcón-González, M. Hey, U. Baumann, A. Huarte, T. Liebscher, C. Bennett, R. English, N. Neben, and A. R. Macís. Evaluation of a transimpedance matrix algorithm to detect anomalous cochlear implant electrode position. *Audiol. Neurotol.* 27(5):347–355, 2022. 10.1159/000523784 35306487

[60] Hosoya M, Fujioka M, Sone T, Okamoto S, Akamatsu W, Ukai H, Ueda HR, Ogawa K, Matsunaga T, Okano H (2017). Cochlear cell modeling using disease-specific iPSCs unveils a degenerative phenotype and suggests treatments for congenital progressive hearing loss. Cell Reports.

[61] Hrncirik F, Roberts IV, Swords C, Christopher PJ, Chhabu A, Gee AH, Bance ML (2022). Impact of scala tympani geometry on insertion forces during implantation. Biosensors.

[62] Hügl S, Rülander K, Lenarz T, Majdani O, Rau TS (2018). Investigation of ultra-low insertion speeds in an inelastic artificial cochlear model using custom-made cochlear implant electrodes. European Archives of Oto-Rhino-Laryngology.

[63] Inaoka T, Shintaku H, Nakagawa T, Kawano S, Ogita H, Sakamoto T, Hamanishi S, Wada H, Ito J (2011). Piezoelectric materials mimic the function of the cochlear sensory epithelium. Proceedings of the National Academy of Sciences.

[64] Incerti PV, Ching TYC, Cowan R (2013). A systematic review of electric-acoustic stimulation: device fitting ranges, outcomes, and clinical fitting practices. Trends in Amplification.

[65] İlik B, Koyuncuoğlu A, Şardan-Sukas Ö, Külah H (2018). Thin film piezoelectric acoustic transducer for fully implantable cochlear implants. Sensors and Actuators A: Physical.

[66] Irving S, Gillespie L, Richardson R, Rowe D, Fallon JB, Wise AK (2014). Electroacoustic stimulation: now and into the future. BioMed Research International.

[67] Ishii T, Takayama M, Takahashi Y (1995). Mechanical properties of human round window, basilar and reissner's membranes. Acta Oto-Laryngologica.

[68] Jain S, Gaurkar S, Deshmukh PT, Khatri M, Kalambe S, Lakhotia P, Chandravanshi D, Disawal A (2019). Applied anatomy of round window and adjacent structures of tympanum related to cochlear implantation. Brazilian Journal of Otorhinolaryngology.

[69] Jang J, Lee J, Jang JH, Choi H (2016). A triboelectric-based artificial basilar membrane to mimic cochlear tonotopy. Advanced Healthcare Materials.

[70] Jang J, Lee J, Woo S, Sly DJ, Campbell LJ, Cho J-H, O'Leary SJ, Park M-H, Han S, Choi J-W, Jang JH, Choi H (2015). A microelectromechanical system artificial basilar membrane based on a piezoelectric cantilever array and its characterization using an animal model. Scientific Reports.

[71] Jeong M, O'Reilly M, Kirkwood NK, Al-Aama J, Lako M, Kros CJ, Armstrong L (2018). Generating inner ear organoids containing putative cochlear hair cells from human pluripotent stem cells. Cell Death Disease.

[72] Jiang C, de Rijk SR, Malliaras GG, Bance ML (2020). Electrochemical impedance spectroscopy of human cochleas for modeling cochlear implant electrical stimulus spread. APL Materials.

[73] Johnson, L. A., C. C. Della Santina, and X. Wang. Temporal bone characterization and cochlear implant feasibility in the common marmoset (*Callithrix jacchus*). *Hear. Res.* 290(1):37–44, 2012. 10.1016/j.heares.2012.05.002 PMC3394878 22583919

[74] Jwair S, Prins A, Wegner I, Stokroos RJ, Versnel H, Thomeer HGXM (2021). Scalar translocation comparison between lateral wall and perimodiolar cochlear implant arrays -a meta-analysis. The Laryngoscope.

[75] Jwair S, Versnel H, Stokroos RJ, Thomeer HGXM (2022). The effect of the surgical approach and cochlear implant electrode on the structural integrity of the cochlea in human temporal bones. Scientific Reports.

[76] Kalkman, R. K., J. J. Briaire, and J. H. M. Frijns. Stimulation strategies and electrode design in computational models of the electrically stimulated cochlea: an overview of existing literature. *Network* 27(2):107–134, 2016. 10.3109/0954898X.2016.1171412 27135951

[77] Kaufmann CR, Henslee AM, Claussen A, Hansen MR (2020). Evaluation of insertion forces and cochlea trauma following robotics-assisted cochlear implant electrode array insertion. Otology Neurotology.

[78] Keppeler D, Kampshoff CA, Thirumalai A, Duque-Afonso CJ, Schaeper JJ, Quilitz T, Töpperwien M, Vogl C, Hessler R, Meyer A, Salditt T, Moser T (2021). Multiscale photonic imaging of the native and implanted cochlea. Proceedings of the National Academy of Sciences.

[79] Kha HN, Chen BK (2006). Determination of frictional conditions between electrode array and endosteum lining for use in cochlear implant models. Journal of Biomechanics.

[80] King J, Shehu I, Roland JT, Svirsky MA, Froemke RC (2016). A physiological and behavioral system for hearing restoration with cochlear implants. Journal of Neurophysiology.

[81] Knoll RM, Trakimas DR, Wu MJ, Lubner RJ, Nadol JB, Ishiyama A, Santos F, Jung DH, Remenschneider AK, Kozin ED (2022). Intracochlear new fibro-ossification and neuronal degeneration following cochlear implant electrode translocation: long-term histopathological findings in humans. Otology Neurotology.

[82] Kobler J-P, Dhanasingh A, Kiran R, Jolly C, Ortmaier T (2015). Cochlear dummy electrodes for insertion training and research purposes: fabrication, mechanical char acterization, and experimental validation. BioMed Research International.

[83] Koch RW, Ladak HM, Elfarnawany M, Agrawal SK (2017). Measuring cochlear duct length - a historical analysis of methods and results. Journal of Otolaryngology Head Neck Surgery.

[84] Koehler KR, Nie J, Longworth-Mills E, Liu X-P, Lee J, Holt JR, Hashino E (2017). Generation of inner ear organoids containing functional hair cells from human pluripotent stem cells. Nature Biotechnology.

[85] Kontorinis G, Lenarz T, Stöver T, Paasche G (2011). Impact of the insertion speed of cochlear implant electrodes on the insertion forces. Otology Neurotology.

[86] Kretzmer EA, Meltzer NE, Haenggeli C-A, Ryugo DK (2004). An animal model for cochlear implants. Archives of Otolaryngology-Head Neck Surgery.

[87] Leblans M, Sismono F, Vanpoucke F, van Dinther J, Lerut B, Kuhweide R, Offeciers E, Zarowski A (2022). Novel impedance measures as biomarker for intracochlear fibrosis. Hearing Research.

[88] Lei IM, Jiang C, Lei CL, de Rijk SR, Tam YC, Swords C, Sutcliffe MPF, Malliaras GG, Bance M, Huang YYS (2021). 3d printed biomimetic cochleae and machine learning co-modelling provides clinical informatics for cochlear implant patients. Nature Communications.

[89] Leon L, Cavilla MS, Doran MB, Warren FM, Abbott JJ (2014). Scala-tympani phantom with cochleostomy and round-window openings for cochlear-implant insertion experiments. Journal of Medical Devices.

[90] Liu W, Atturo F, Aldaya R, Santi P, Cureoglu S, Obwegeser S, Glueckert R, Pfaller K, Schrott-Fischer A, Rask-Andersen H (2015). Macromolecular organization and fine structure of the human basilar membrane -relevance for cochlear implantation. Cell and Tissue Research.

[91] Liu Y, Zhu Y, Liu J, Zhang Y, Liu J, Zhai J (2018). Design of bionic cochlear basilar membrane acoustic sensor for frequency selectivity based on film triboelectric nanogenerator. Nanoscale Research Letters.

[92] Lu W, Xu J, Shepherd RK (2005). Cochlear implantation in rats: a new surgical approach. Hearing Research.

[93] Majdani O, Schurzig D, Hussong A, Rau T, Wittkopf J, Lenarz T, Labadie RF (2010). Force measurement of insertion of cochlear implant electrode arrays in vitro: comparison of surgeon to automated insertion tool. Acta Oto-Laryngologica.

[94] Malherbe TK, Hanekom T, Hanekom JJ (2015). The effect of the resistive properties of bone on neural excitation and electric fields in cochlear implant models. Hearing Research.

[95] Mancheño M, Aristegui M, Sañudo JR (2017). Round and oval window anatomic variability: its implication for the vibroplasty technique. Otology Neurotology.

[96] Manoussaki D, Chadwick RS, Ketten DR, Arruda J, Dimitriadis EK, O'Malley JT (2008). The influence of cochlear shape on low-frequency hearing. Proceedings of the National Academy of Sciences of the United States of America.

[97] Marx M, Girard P, Escudé B, Barone P, Fraysse B, Deguine O (2013). Cochlear implantation feasibility in rhesus macaque monkey: anatomic and radiologic results. Otology Neurotology.

[98] Meas, S. J., K. Nishimura, M. Scheibinger, and A. Dabdoub. In vitro methods to cultivate spiral ganglion cells, and purification of cellular subtypes for induced neuronal reprogramming. *Front. Neurosci.* 12: 822, 2018. 10.3389/fnins.2018.00822 PMC6249511 30498430

[99] Meenderink SWF, Shera CA, Valero MD, Liberman MC, Abdala C (2019). Morphological immaturity of the neonatal organ of corti and associated structures in humans. Journal of the Association for Research in Otolaryngology.

[100] Mellott AJ, Shinogle HE, Nelson-Brantley JG, Detamore MS, Staecker H (2017). Exploiting decellularized cochleae as scaffolds for inner ear tissue engineering. Stem Cell Research Therapy.

[101] Meng J, Li S, Zhang F, Li Q, Qin Z (2016). Cochlear size and shape variability and implications in cochlear implantation surgery. Otology Neurotology: Official Publication of the American Otological Society, American Neurotology Society and European Academy of Otology and Neurotology.

[102] Mittmann M, Ernst A, Mittmann P, Todt I (2017). Insertional depth-dependent intracochlear pressure changes in a model of cochlear implantation. Acta Oto-Laryngologica.

[103] Mittmann P, Ernst A, Todt I (2014). Intracochlear pressure changes due to round window opening: a model experiment. The Scientific World Journal.

[104] Mittmann, P., G. Lauer, A. Ernst, S. Mutze, F. Hassepass, S. Arndt, D. Arweiler-Harbeck, and F. Christov. Electrophysiological detection of electrode fold-over in perimodiolar cochlear implant electrode arrays: a multi-center study case series. *Eur. Arch. Oto-rhino-laryngol.* 277(1):31–35, 2020. 10.1007/s00405-019-05653-9 31552525

[105] Mittmann P, Mittmann M, Ernst A, Todt I (2017). Intracochlear pressure changes due to 2 electrode types: an artificial model experiment. Otolaryngology-Head and Neck Surgery.

[106] Mu X, Bertron T, Dunn C, Qiao H, Wu J, Zhao Z, Saldana C, Qi HJ (2017). Porous polymeric materials by 3d printing of photocurable resin. Materials Horizons.

[107] Mukherjee N, Roseman RD, Willging JP (2000). The piezoelectric cochlear implant: concept, feasibility, challenges, and issues. Journal of Biomedical Materials Research.

[108] Narasimhan, N., K. E. Riojas, T. L. Bruns, J. E. Mitchell, R. J. Webster, and R. F. Labadie. A simple manual roller wheel insertion tool for electrode array insertion in minimally invasive cochlear implant surgery. In 2019 Design of Medical Devices Conference, V001T06A003, Minneapolis, MN, USA, 2019.

[109] Nguyen Y, Bernardeschi D, Kazmitcheff G, Miroir M, Vauchel T, Ferrary E, Sterkers O (2015). Effect of embedded dexamethasone in cochlear implant array on insertion forces in an artificial model of scala tympani. Effect of embedded dexamethasone in cochlear implant array on insertion forces in an artificial model of scala tympani: Otology Neurotology.

[110] Ni G, Elliott SJ, Ayat M, Teal PD (2014). Modelling cochlear mechanics. BioMed Research International.

[111] Nordfalk KF, Rasmussen K, Hopp E, Greisiger R, Jablonski GE (2014). Scalar position in cochlear implant surgery and outcome in residual hearing and the vestibular system. International Journal of Audiology.

[112] Patpatiya P, Chaudhary K, Shastri A, Sharma S (2022). A review on polyjet 3d printing of polymers and multi-material structures. Proceedings of the Institution of Mechanical Engineers, Part C: Journal of Mechanical Engineering Science.

[113] Pietsch, M., L. Aguirre Dávila, P. Erfurt, E. Avci, T. Lenarz, and A. Kral. Spiral form of the human cochlea results from spatial constraints. *Sci. Rep.* 7(1):7500, 2017. 10.1038/s41598-017-07795-4 PMC5548794 28790422

[114] Pile J, Sweeney AD, Kumar S, Simaan N, Wanna GB (2017). Detection of modiolar proximity through bipolar impedance measurements: bipolar electrical impedance. The Laryngoscope.

[115] Potrusil, T., A. Heshmat, S. Sajedi, C. Wenger, L. Johnson Chacko, R. Glueckert, A. Schrott-Fischer, and F. Rattay. Finite element analysis and three-dimensional reconstruction of tonotopically aligned human auditory fiber pathways: a computational environment for modeling electrical stimulation by a cochlear implant based on micro-CT. *Hear. Res.* 393:108001, 2020. 10.1016/j.heares.2020.108001 32535276

[116] Prendergast ME, Burdick JA (2020). Recent advances in enabling technologies in 3d printing for precision medicine. Advanced Materials.

[117] Radotiá V, Bedalov A, Drviš P, Braeken D, Kovačić D (2019). Guided growth with aligned neurites in adult spiral ganglion neurons cultured in vitro on silicon micro-pillar substrates. Journal of Neural Engineering.

[118] Rattay, F., R. N. Leao, and H. Felix. A model of the electrically excited human cochlear neuron. II. influence of the three-dimensional cochlear structure on neural excitability. *Hear. Res.* 153(1):64–79, 2001. 10.1016/s0378-5955(00)00257-4 11223297

[119] Rattay F, Potrusil T, Wenger C, Wise AK, Glueckert R, Schrott-Fischer A (2013). Impact of morphometry, myelinization and synaptic current strength on spike conduction in human and cat spiral ganglion neurons. PLoS ONE.

[120] Raufer, S., C. Idoff, A. Zosuls, G. Marino, N. Blanke, I. J. Bigio, J. T. O'Malley, B. J. Burgess, J. B. Nadol, J. J. Guinan, and H. H. Nakajima. Anatomy of the human osseous spiral lamina and cochlear partition bridge: relevance for cochlear partition motion. *J. Assoc. Res. Otolaryngol.* 21(2):171–182, 2020. 10.1007/s10162-020-00748-1 PMC7270316 32166603

[121] Rebscher SJ, Hetherington AM, Snyder RL, Leake PA, Bonham BH (2007). Design and fabrication of multichannel cochlear implants for animal research. Journal of Neuroscience Methods.

[122] Rebscher SJ, Talbot N, Bruszewski W, Heilmann M, Brasell J, Merzenich MM (1996). A transparent model of the human scala tympani cavity. Journal of Neuroscience Methods.

[123] Reiss LA, Kirk J, Claussen AD, Fallon JB (2022). Animal models of hearing loss after cochlear implantation and electrical stimulation. Hearing Research.

[124] Roland JT (2005). A model for cochlear implant electrode insertion and force evaluation: results with a new electrode design and insertion technique. The Laryngoscope.

[125] Roland PS, Wright CG (2006). Surgical aspects of cochlear implantation: mechanisms of insertional trauma. Cochlear and Brainstem Implants.

[126] Sahni RS, Paparella MM, Schachern PA, Goycoolea MV, Le CT (1987). Thickness of the human round window membrane in different forms of otitis media. Archives of Otolaryngology -Head and Neck Surgery.

[127] Salkim, E., M. Zamani, D. Jiang, S. R. Saeed, and A. Demosthenous. Insertion guidance based on impedance measurements of a cochlear electrode array. *Front. Comput. Neurosci.* 16:862126, 2022. 10.3389/fncom.2022.862126 PMC9260075 35814346

[128] Schnabl J, Glueckert R, Feuchtner G, Recheis W, Potrusil T, Kuhn V, Wolf-Magele A, Riechelmann H, Sprinzl GM (2012). Sheep as a large animal model for middle and inner ear implantable hearing devices: a feasibility study in cadavers. Otology Neurotology: Official Publication of the American Otological Society, American Neurotology Society and European Academy of Otology and Neurotology.

[129] Schuster D, Kratchman LB, Labadie RF (2015). Characterization of intracochlear rupture forces in fresh human cadaveric cochleae. Otology Neurotology.

[130] Seliet A, El Hamshary A, El Refai A, Ali A, Gabal S (2018). Human round window: morphometry and topographical anatomy and their effect on cochlear implantation. Benha Medical Journal.

[131] Simoni, E., E. Gentilin, M. Candito, G. Borile, F. Romanato, M. Chicca, S. Nordio, M. Aspidistria, A. Martini, D. Cazzador, and L. Astolfi. Immune response after cochlear implantation. *Front. Neurol.*, 2020. 10.3389/fneur.2020.00341 10.3389/fneur.2020.00341 PMC7240074 32477241

[132] Singla A, Sahni D, Gupta A, Loukas M, Aggarwal A (2014). Surgical anatomy of round window and its implications for cochlear implantation: surgical anatomy of round window. Clinical Anatomy.

[133] Singla A, Sahni D, Gupta AK, Aggarwal A, Gupta T (2015). Surgical anatomy of the basal turn of the human cochlea as pertaining to cochlear implantation. Otology Neurotology.

[134] Smit JE, Hanekom T, van Wieringen A, Wouters J, Hanekom JJ (2010). Threshold predictions of different pulse shapes using a human auditory nerve fibre model containing persistent sodium and slow potassium currents. Hearing Research.

[135] Souza P (2004). Compression: from cochlea to cochlear implants. Ear and Hearing.

[136] Spitzer ER, Waltzman SB, Landsberger DM, Friedmann DR (2021). Acceptance and benefits of electro-acoustic stimulation for conventional-length electrode arrays. Audiology and Neurotology.

[137] Su WY, Marion MS, Hinojosa R, Matz GJ (1982). Anatomical measurements of the cochlear aqueduct, round window membrane, round window niche, and facial recess. The Laryngoscope.

[138] Swaddiwudhipong N, Jiang C, Landry TG, Bance M (2021). Investigating the electrical properties of different cochlear implants. Otology Neurotology.

[139] Tabibi S, Boulet J, Dillier N, Bruce IC (2021). Phenomenological model of auditory nerve population responses to cochlear implant stimulation. Journal of Neuroscience Methods.

[140] Takahashi M, Arai Y, Sakuma N, Yabuki K, Sano D, Nishimura G, Oridate N, Usami S-I (2018). Cochlear volume as a predictive factor for residual-hearing preservation after conventional cochlear implantation. Acta Oto-Laryngologica.

[141] Takanen, M., I. C. Bruce, and B. U. Seeber. Phenomenological modelling of electrically stimulated auditory nerve fibers: a review. *Network* 27(2):157–185, 2016. 10.1080/0954898X.2016.1219412 27573993

[142] Takanen M, Seeber BU (2022). A phenomenological model reproducing temporal response characteristics of an electrically stimulated auditory nerve fiber. Trends in Hearing.

[143] Tissue engineering—latest research and news—nature. https://www.nature.com/subjects/tissue-engineering. Visited 19 Nov 2020.

[144] Todd C, Naghdy F, Svehla M (2007). Force application during cochlear implant insertion: an analysis for improvement of surgeon technique. IEEE Transactions on Biomedical Engineering.

[145] Trevino M, Lobarinas E, Maulden AC, Heinz MG (2019). The chinchilla animal model for hearing science and noise-induced hearing loss. The Journal of the Acoustical Society of America.

[146] Tsuji, T., A. Nakayama, H. Yamazaki, and S. Kawano. Artificial cochlear sensory epithelium with functions of outer hair cells mimicked using feedback electrical stimuli. *Micromachines* 9(6):273, 2018. 10.3390/mi9060273 PMC6187550 30424206

[147] Usevitch DE, Park AH, Scheper V, Abbott JJ (2021). Estimating the pose of a guinea-pig cochlea without medical imaging. Otology Neurotology.

[148] Vanpoucke F, Zarowski A, Casselman J, Frijns J, Peeters S (2004). The facial nerve canal: an important cochlear conduction path revealed by clarion electrical field imaging. Otology Neurotology.

[149] Von Békésy G, Peake WT (1990). Experiments in Hearing. The Journal of the Acoustical Society of America.

[150] Wanna, G. B., J. H. Noble, R. H. Gifford, M. S. Dietrich, A. D. Sweeney, D. Zhang, B. M. Dawant, A. Rivas, and R. F. Labadie. Impact of intrascalar electrode location, electrode type, and angular insertion depth on residual hearing in cochlear implant patients: preliminary results. *Otol. Neurotol.* 36(8):6, 2015. 10.1097/MAO.0000000000000829 PMC7187917 26176556

[151] West CD (1985). The relationship of the spiral turns of the cochlea and the length of the basilar membrane to the range of audible frequencies in ground dwelling mammals. The Journal of the Acoustical Society of America.

[152] Wever EG (1938). the width of the basilar membrane in man. Annals of Otology, Rhinology Laryngology.

[153] WHO—estimates. WHO. http://www.who.int/deafness/estimates/en/. Visited 28 April 2020.

[154] Wright CG, Roland PS (2013). Vascular trauma during cochlear implantation: a contributor to residual hearing loss?. Otology Neurotology.

[155] Würfel W, Lanfermann H, Lenarz T, Majdani O (2014). Cochlear length determination using cone beam computed tomography in a clinical setting. Hearing Research.

[156] Yi H, Guo W, Chen W, Chen L, Ye J, Yang S (2016). Miniature pigs: a large animal model of cochlear implantation. American Journal of Translational Research.

[157] Yoo S, Wang G, Rubinstein J, Vannier M (2000). Three-dimensional geometric modeling of the cochlea using helico-spiral approximation. IEEE Transactions on Biomedical Engineering.

[158] Zhang X, Gan RZ (2013). Dynamic properties of human round window membrane in auditory frequencies. Medical Engineering Physics.

[159] Zhao C, Knisely KE, Colesa DJ, Pfingst BE, Raphael Y, Grosh K (2019). Voltage readout from a piezoelectric intracochlear acoustic transducer implanted in a living guinea pig. Scientific Reports.

